# Laser Sensors for Displacement, Distance and Position

**DOI:** 10.3390/s19081924

**Published:** 2019-04-24

**Authors:** Young Soo Suh

**Affiliations:** School of Electrical Engineering, University of Ulsan, Namgu, Ulsan 44610, Korea; yssuh@ulsan.ac.kr

**Keywords:** laser sensors, distance sensors, displacement sensors, position sensors, optical sensors

## Abstract

Laser sensors can be used to measure distances to objects and their related parameters (displacements, position, surface profiles and velocities). Laser sensors are based on many different optical techniques, such as triangulation, time-of-flight, confocal and interferometric sensors. As laser sensor technology has improved, the size and cost of sensors have decreased, which has led to the widespread use of laser sensors in many areas. In addition to traditional manufacturing industry applications, laser sensors are increasingly used in robotics, surveillance, autonomous driving and biomedical areas. This paper outlines some of the recent efforts made towards laser sensors for displacement, distance and position.

## 1. Contributions

This special issue focuses on recent research results in the area of laser sensors for distance and its related parameters: 14 papers are accepted after the review.

The papers can be classified based on sensors’ measurement principles: Triangulation sensor [1,2,3,4], time-of-flight sensor [5,6,7], confocal sensor [8], interferometric sensor [9], fiber Bragg grating sensor [10], and laser Doppler velocimetry [11]. There are also three papers [12,13,14], which are not classified in the above categories.

In the following, a brief overview of each paper is given.

### 1.1. Triangulation Sensor

Laser triangulation sensor consists of a laser light source and a detector. The laser beam is projected on the target and the reflected signal is detected by a detector, where an image sensor is usually used.

In [1], angle change of airfoils inside a wind tunnel is measured using four commercial triangulation sensors (Acuity AR700-32). Small changes in this angle (called the angle of attack) can create significant changes in the forces and moments, so accurately measuring the angle of attack is critical. The four sensors measure the distance between the wind tunnel walls and the airfoil, which was then used to calculate the model position. The proposed system in [1] is able to detect model deflection and rotation that would otherwise not have been detected by other sensors. Since the measured distance tends to drift slightly with temperature, a temperature compensation algorithm is proposed.

In [2], valve spool displacement of a direct acting solenoid on-off valve is measured using a commercial laser triangulation sensor. The main objective is to derive an electromagnetic model using the calculated inductance of an on-off solenoid to solve for the varying air gap. To obtain this model, coil current is measured using a sensing electrical resistor and air gap is measured using a laser triangulation sensor. Once the model is derived, an air gap of an on-off solenoid can be predicted without using a laser sensor. The derived model can predict the transition of an air gap in atmosphere with an error under ±7%.

In [3], 3D surface maps of turbine blades are expressed in the robot coordinate using a specially built laser triangulation sensor unit mounted on a robot arm. The sensor unit consists of two line lasers and a CMOS camera. The main contribution of this paper is the calibration algorithm of the sensor unit. Due to the large nonlinearities present in a camera and laser diodes, large range distances become difficult to measure with high precision. To improve accuracy, a calibration model is proposed that involves the parameters of the camera, lens, laser positions, and sensor position on the robot arm related to the robot base to find the best accuracy in the distance range of the application. Results showed an average accuracy of 0.3 mm on a displacement of approximately 180 mm.

In [4], position of prismatic–revolute–revolute (three links with each link consisting of a prismatic pair and two rotating pairs) parallel platform is measured using three laser triangulation sensors and linear grating encoders. The objective is to develop a three-degrees-of-freedom full closed-loop control precision tracking system. Equally-spaced laser displacement sensors and linear grating encoders were used in combination not only for measurement but also for feedback control.

### 1.2. Time-of-Flight Sensor

Time-of-flight sensor measures distance by sending a narrow laser beam towards the object and receiving the reflected signal. A short pulse could be used to measure a round-trip time. Also, modulated continuous light could be used, where phase shift of the reflected signal is measured.

In [5], the distance precision of the commercial 2D laser scanner (Z+F Profiler 9012A) is analyzed. In the close range, divergent incident laser light can lead to a reduction of the signal input power deteriorating both the backscattered intensity and the precision of the distance measurements. To analyze the intensity-dependent distance precision, static scanning of surfaces with different backscatter is investigated. Based on this investigation, intensity-dependent stochastic models are derived. These models can be used for quality assurance and deformation analysis. In addition, they could be used in the system calibration leading to better estimates for the calibration parameters.

In [6], a small drone detection problem using 3D LIDAR is considered. Instead of building a physical system, an augmented dataset is proposed to generate a virtual target considering the laser beam and scanning characteristics. Thus design and testing of drone detection algorithm can be done without real sensor data. Also, a drone detection algorithm is proposed using voxel-based background subtraction and variable radially bounded nearest neighbor (V-RBNN) method. The proposed detection algorithm is tested using an augmented datasets.

In [7], a new extrinsic parameter calibration method is proposed for multiple laser range finders. When multiple laser sensors are used, it is important to know the relative pose of each sensor. In this paper, a simple calibration algorithm is proposed, where a common cuboid-shaped corridor is used as the experiment environment. Since the corridor is very common in indoor building, multiple laser sensors can be calibrated without using special artificial target in the environment and supervised data association.

### 1.3. Confocal Sensor

In confocal sensors, laser light is projected on the target and light reflected from the target is detected through aperture. When the target is on the focal plane, the reflected light intensity becomes maximum. Using this property, the distance to target can be measured very accurately (for example 0.01 μm resolution at the target distance 6 mm for a sensor used in [8]).

In [8], surface topographic feature such as roughness is measured using a commercial laser confocal microscopy (Keyenece LT-9010M). The main focus is to mount the sensor onto a robotic arm and to measure surface features in-situ instead of in a lab environment. The calibration of the sensor unit is proposed to reduce scattering noise at steep angles and background noise for specular reflection. Experimental data shows that the proposed system is able to measure surfaces with Ra from 0.2–7 μm, which covers a common range of milling, turning and grinding.

### 1.4. Interferometric Sensor

In interferometric sensor, laser beam is projected both onto the target and the reference mirror. The two reflected signals (from the target and the reference mirror) are superimposed, causing the phenomenon of interference. Theoretically the sensor can measure subwavelength (nanometer range) displacements.

In [9], a very small displacement of the mass–spring system is measured using laser interferometry. The objective is to estimate acceleration from mass displacement; that is, interferometric optomechanical accelerometer is proposed. To overcome the narrow non-ambiguity range of the interferometry, a novel measurement method based on synthetic wavelength and single wavelength superheterodyne interferometry is proposed and the prototype is constructed. The results prove an estimated acceleration measurement resolution of around 10 μg and a non-ambiguity range larger than 200 mg, which is more than 100 times that of the single wavelength-based optical accelerometer.

### 1.5. Fiber Bragg Grating Sensor

An optical fiber with special periodic gratings (fiber Bragg grating) reflects particular wavelength of light and transmits all others. If this fiber is stretched or strained, the reflected wavelength changes. Using this property, fiber Bragg grating (FBG) can be used as displacement sensors. Unlike other laser sensors, FBG is not a non-contact sensor.

In [10], acoustic emissions (transient elastic waves within a material caused by the rapid release of localized stress energy) is measured using FBG. Existing FBGs have a relatively small measurement frequency bandwidth and the strain sensitivity is not sufficient to measure the acoustic emissions induced events. By combing self-mixing interference and FBG technology, a new compact and cost-effective system with a wide dynamic measurement range is proposed.

### 1.6. Laser Doppler Velocimeter

When the laser is aimed at a target, a portion of light reflects back into the laser where it mixes with the strong laser field. When the movement of the object has a component along the direction of the laser beam, the phase of the reflected light continuously shifts with respect to the original laser light, resulting in a periodic variation of the feedback into the laser at a frequency equal to the Doppler frequency. From this Doppler frequency, the velocity of the target can be derived.

In [11], six degrees of freedom motion is estimated using multiview laser Doppler speed sensing. The accuracy of this system depends on calibration precision. In contrast with the conventional method where only laser geometry is independently calibrated from images, the proposed method simultaneously optimizes all laser parameters and directly associates them with the motion-sensing model.

### 1.7. Miscellaneous Sensors

In [12], optical target (high-index glass ball retroreflector) is used to measure lateral positions. A laser is projected into the glass ball and its reflection is acquired by a camera. An image processing algorithm is proposed to detect lateral positions of the glass ball.

In [13], a numerical simulation platform is proposed for a Hartmann–Shack wavefront sensor. The simulation platform contains every step in the wavefront detection and reconstruction process, from the generation of the input wavefronts to the computation of the reconstructed wavefronts and their respective resulting errors. This platform can be used for designing the sensor, especially when a custom focal-plane chip is intended.

In [14], a fast steering mirror (FSM) is used to improve laser point stability. The laser source drift contains two DOF angular errors and two DOF displacement errors. In this paper, a new FSM compensation system with double Porro prisms to reduce the 4-DOF laser errors of the laser source is proposed. The experiment results show that the proposed system can eliminate 97 % of the laser errors.
